# Malignant Pericardial Effusion Presenting as a Sequela of Lung Adenocarcinoma

**DOI:** 10.7759/cureus.57287

**Published:** 2024-03-30

**Authors:** Nivedha Balaji, Gabriel Velez Oquendo, Moyan Sun, Christine Sykalo, Imran Khan

**Affiliations:** 1 Internal Medicine, Northeast Georgia Medical Center Gainesville, Gainesville, USA; 2 Cardiology, Northeast Georgia Medical Center Gainesville, Gainesville, USA

**Keywords:** pericardiectomy, pericardiotomy, pericardiocentesis, lung adenocarcinoma, malignant pericardial effusion, pericardial effusion

## Abstract

Pericardial effusion is a collection of fluid in the pericardial sac that can result in symptoms such as shortness of breath, pleuritic chest pain, and/or hemodynamic instability. Malignant pleural effusions are seen in a few cancer patients and are associated with poor prognosis. Here, we present the case of a 65-year-old female with a large malignant pericardial effusion in the setting of advanced-stage lung adenocarcinoma.

## Introduction

Pericardial effusions are described as the accumulation of fluid within the pericardial sac, separating the visceral and parietal layers of the heart [1,2]. The pericardial sac or cavity normally contains 50-100 mL of fluid. This cavity can accommodate over 1000 mL without cardiac compromise when accumulated slowly [3]. Major causes of pericardial effusion include infection, malignancy, heart failure, renal failure, liver cirrhosis, autoimmune disease, Dressler syndrome, and medications such as hydralazine and procainamide [1]. Primary pericardial neoplasms are 40x less common than metastatic pericardial effusions [3]. The most common causes of pericardial effusions are lung and breast cancers, leading to effusions via local lymphatic invasion, direct mediastinal invasion, and less commonly through hematogenous invasion [1,2,4]. We present the case of a middle-aged female with a large malignant pericardial effusion in the setting of advanced-stage lung adenocarcinoma. 

## Case presentation

Our patient was a 65-year-old female with a past medical history of previously treated bladder cancer, tobacco use disorder, gross hematuria on chronic indwelling Foley catheter, and ulcerative colitis. She presented to her primary care physician's office with complaints of persistent cough despite a trial of antibiotic therapy and symptomatic treatment. She was referred to a pulmonologist's office for distressing chronic cough, dyspnea on exertion, and 10 lbs. weight loss present for a few months. A computed tomography (CT) scan of her chest showed bulky left hilar lymphadenopathy measuring 4.7 x 3.1 cm with narrowing of the left upper lobe with a contiguous left lower lobe thick-walled cavitary lesion measuring 5.7 x 4.4 cm and a pulmonary nodule in the right lower lobe measuring 6 mm. Bronchoscopy with endobronchial biopsy was performed which showed an infiltrating mass of moderately differentiated adenocarcinoma of the left upper lobe that was TTF-1 negative and PD-L1 strongly positive at 80%. Subsequent positron emission tomography (PET) and CT showed a left pulmonary mass extending to the left hilum with widespread metastatic disease to the bones, liver, soft tissues, left adrenal gland, and apex of the heart in the mitral valve and right ventricular region. She was then referred to radiation oncology for palliative radiation and immunotherapy with pembrolizumab. During treatment, the patient then developed a large left-sided pleural effusion that was treated with the placement of a pleural catheter system. 

**Figure 1:**
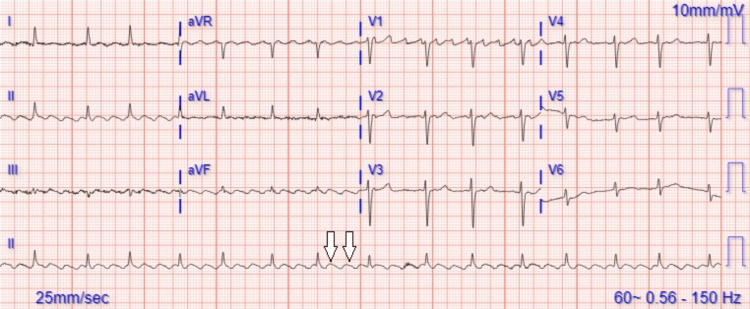
Electrocardiogram showing typical atrial flutter with variable atrioventricular conduction without electrical alternans. The white arrows indicate the typical atrial flutter pattern.

**Figure 2:**
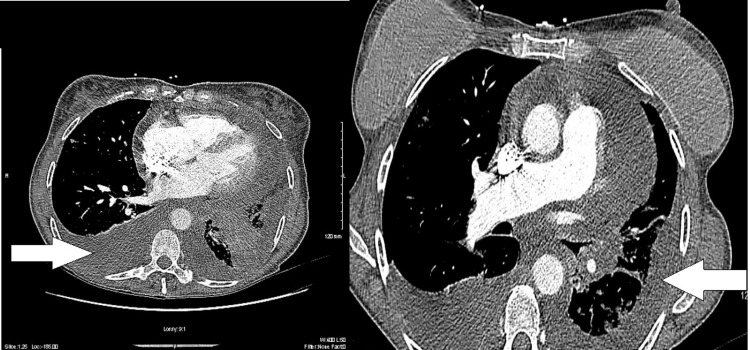
CT chest showing bilateral pleural effusions, loculations, and a moderate-sized pericardial effusion.

**Figure 3:**
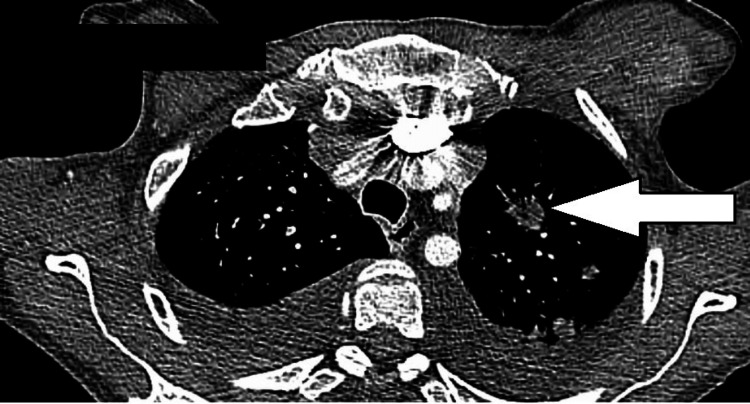
Left-sided pulmonary nodule noted on CT chest.

The patient was readmitted several weeks later with symptomatic tachycardia and new-onset atrial flutter without electrical alternans (Figure 1). Workup with chest X-ray showed new bilateral pleural effusions and a CT angiogram of the lungs revealed bilateral small distal subsegmental pulmonary emboli, known left lower lobe cavitary mass, local metastatic left hilar lymphadenopathy, and a moderate to large pericardial effusion without signs of cardiac tamponade (Figures 2, 3). Due to her worsening condition, care was escalated to the intensive care unit. 

**Figure 4:**
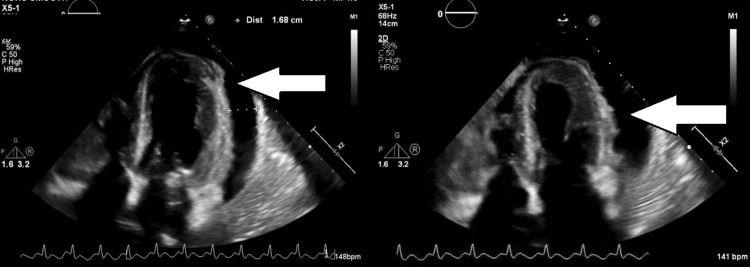
Four-chamber view of the echocardiogram showing a moderate-sized pericardial effusion, and a left ventricular apical anterior myocardium with Echo density suspicious for cardiac mass. Arrows are pointing towards the pericardial effusion

Significant laboratory values are listed in Table 1. For the new-onset atrial flutter, she was started on amiodarone and heparin infusions, with piperacillin-tazobactam antibiotic treatment due to leukocytosis. The echocardiogram showed a left ventricular ejection fraction of 40-45%, mild global hypokinesis of the left ventricle with moderate concentric hypertrophy, a right ventricular systolic pressure 76 mmHg, mildly dilated right atrium, mild-to-moderate mitral valve regurgitation, moderate to severe tricuspid valve regurgitation, mild pulmonic valve regurgitation, and a moderate to large pericardial effusion without evidence of cardiac tamponade (Figures 4A, 4B). 

**Table 1:** Laboratory testing performed on admission.

Component	Patient’s value	Normal range
Sodium	120 mmol/L	135-148 mmol/L
Chloride	87 mmol/L	100-110 mmol/L
Hemoglobin	11.4 g/dL	12-16 g/dL
White blood cells	19.2 K/uL	4.8-10.8 K/uL
International normalized ratio (INR)	1.35	0.85-1.13
Urine sodium	<10 mmol/L	40-220 mmol/L
Urine osmolality	682 mosm/KgH2O	500 - 800 mosm/kgH2O
Serum osmolality	248 mosm/kg	275-295 mosm/kg
Thyroid-stimulating hormone (TSH)	8.395 uIU/mL	0.34-4.82 uIU/mL
Free thyroxine (T4)	1.14 ng/dL	0.71-1.9 ng/dL
Alkaline phosphatase	275 U/L	45-136 U/L
N-terminal prohormone of brain natriuretic peptide (NT pro-BNP)	2838 pg/mL	<900 pg/mL
Lactate dehydrogenase	597 U/L	84-246 U/L
Total protein	6 g/dL	6-8.3 g/dL

Throughout the patient’s hospital course, a transesophageal echocardiogram was considered to further evaluate the left ventricular mass, but it was not pursued because the patient was deemed a high-risk candidate. Instead, cardiac magnetic resonance imaging was planned in the outpatient setting to investigate cardiac metastasis. In the meantime, 800 cc of serosanguinous fluid was drained via thoracentesis with the fluid study results indicating an exudative process listed in Table 2. Pathology results revealed focal malignant cells consistent with non-small cell carcinoma and numerous reactive mesothelial cells. Cardiothoracic surgery subsequently performed left anterior lateral mini-thoracotomy with pericardial window and drain placement and left tube thoracostomy, with cytopathology results showing invasive non-small cell carcinoma involving dense fibroconnective tissue of the pericardium and malignant cells compatible with non-small cell carcinoma of lung origin. Subsequently, her pigtail catheter and pericardial window were removed. About five weeks later, she returned to the hospital with worsening left-sided loculated pleural effusion which was complicated by pneumonia. Immunotherapy and chemotherapy were withheld at that time. However, the patient had goals of care discussion with palliative medicine, and it was decided to pursue non-curative/palliative care. 

**Table 2:** Fluid studies from thoracentesis

Component	Patient’s value
Glucose	88 mg/dL
Hematocrit	0.3%
Culture	No growth
Red blood cells	31,000
White blood cells	2587/mm3
Segments	22%
Lymphocytes	30%
Monocytes	16%
Mesothelial	13%
Protein	2.2 gm/dL
Lactate dehydrogenase	294 U/L

## Discussion

Malignant pericardial effusions are seen in about 10% of patients diagnosed with cancer [5]. Primary malignant pericardial effusions due to myxomas, lipomas, fibrosarcoma, lymphangiomas, hemangiomas, and neurofibromas are less common than secondary malignant pericardial effusions due to metastatic disease. Metastatic pericardial effusions can be attributed to lung adenocarcinoma or squamous cell carcinoma in up to 50% of cases, breast cancer in up to 18% of cases, and melanoma or gastrointestinal adenocarcinoma in up to 7% of patients [1,4,6]. They are frequently caused by hematogenous metastasis, lymphatic invasion, direct penetration, or intracavitary diffusion through the inferior vena cava or pulmonary veins [1,5-8]. 

Pericardial effusions clinically present with dyspnea, chest pain or chest pressure, hemodynamic lability, and/or syncope [9]. The clinical presentation of pericardial effusions in the setting of lung cancer is quite rare and is associated with poor outcomes as it is a marker of advanced disease, with 60% of the patients with malignant pericardial effusions developing cardiac tamponade [6]. Cardiac tamponade presents with worsening dyspnea, pulsus paradoxus, and Beck’s triad which entails jugular venous distension, distant heart sounds, and hypotension [4]. Cardiac tamponade can be fatal if appropriate and timely care is not provided [10,11]. It can also present with pericarditis in the setting of neoplastic infiltration that causes scarring and subsequent loss of elasticity of the cardiac tissue. These patients can present with additional symptoms such as sinus tachycardia, hypotension, jugular venous distention, pericardial friction rub, and rarely with Kussmaul respirations, and pulsus paradoxus [3]. 

Although both cardiac tamponade and constrictive pericarditis caused by pericardial effusions are clinical diagnoses, various imaging modalities can assist in assessment and early intervention [3]. Non-specific findings for pericardial effusions include cardiomegaly on chest radiography and electrical alterations or low-amplitude findings on electrocardiography [4]. However, echocardiography remains the gold standard for identifying the size, location, and pathophysiologic impact on the heart [4,12]. The treatment pathway depends on the size of the effusion and a patient's clinical presentation. If a patient is experiencing cardiac tamponade physiology symptoms, emergent pericardiocentesis is warranted [4]. However, if the patient is stable, non-emergent pericardiocentesis, chemotherapy, radiotherapy, and surgical pericardial window may all be considered. Although pericardial windows are associated with a lower risk of recurrence, in patients with malignant pericardial effusions secondary to lung carcinoma pericardial windows are associated with a lower overall survival outcome. Previous studies have shown that systemic chemotherapy used independently and in combination with pericardial window or pericardiocentesis are effective [12]. While balloon pericardiotomy or surgical pericardiectomy are also available options, patients with malignant pericardial effusions tend to undergo pericardial window to prevent re-accumulation [5]. Fluid from the pericardial effusion can then be evaluated by a pathologist for malignant cytology [4]. 

Malignant pericardial effusions are associated with poor prognosis because they are rarely the first metastatic site in cancer, indicating that there is an advanced disease burden [1]. Lung cancer and other solid tumor-induced pericardial effusions are associated with limited survival when compared to breast cancer or hematologic malignancies [3]. Hence, it is essential to include malignancy as part of the differential diagnosis in the setting of rapidly accumulating or recurrent pericardial effusions [6]. 

Additionally, immunotherapy-induced adverse effects from pembrolizumab may also be considered in this patient. The incidence of pembrolizumab-related adverse events includes colitis, pneumonitis, hepatitis, various endocrinopathies, myocarditis, vasculitis, arrhythmias, and cardiac tamponade. While it has been reported that the development of pericardial tamponade from pericardial effusion can take up to 3-4 months, however, the pathophysiology behind the development of pericardial effusion is poorly understood. Immune checkpoint inhibitor-induced pericardial effusion should be a diagnosis of exclusion [13]. This patient’s complications of pleural and pericardial effusions are likely secondary to non-small cell lung carcinoma, but immunotherapy association cannot be excluded. 

## Conclusions

In conclusion, pericardial effusions can arise from various causes such as infection, malignancy, and autoimmune disease; however, neoplastic pericardial effusions are associated with poor prognoses. Delay in recognition of pericardial effusions can result in pericardial tamponade and even fatal outcomes. Hence, it is essential to recognize signs and symptoms of pericardial effusions and cardiac tamponade so patients can be diagnosed and managed in an emergent manner. 
